# Synthetic Lethality of the *bfr* and *mbfA* Genes Reveals a Functional Relationship between Iron Storage and Iron Export in Managing Stress Responses in *Bradyrhizobium japonicum*

**DOI:** 10.1371/journal.pone.0157250

**Published:** 2016-06-10

**Authors:** Siva Sankari, Mark R. O’Brian

**Affiliations:** Department of Biochemistry, State University of New York at Buffalo, Buffalo, New York, United States of America; East Carolina University School of Medicine, UNITED STATES

## Abstract

An *mbfA* mutant of *Bradyrhizobium japonicum* defective in iron export is sensitive to short term exposure to high levels iron or H_2_O_2_. Here, we found that the *mbfA* strain grown in elevated iron media (100 μM) became resistant to those treatments, suggesting a stress response adaptation. The *bfr* gene encodes the iron storage protein bacterioferritin, and its expression is derepressed by iron. An *mbfA bfr* double mutant showed a loss of stress adaptation, and had a severe growth phenotype in high iron media. Moreover, a *bfr*^*up*^ allele in which *bfr* is constitutively derepressed conferred stress tolerance on an *mbfA* mutant without elevating the iron content in the growth media. The intracellular iron content of the *mbfA bfr* double mutant was substantially higher than that found in the wild type, even when grown in relatively low iron media (5 μM). Under that condition, iron-responsive gene expression was aberrant in the *mbfA bfr* strain. Moreover, the double mutant was sensitive to the iron-activated antibiotic streptonigrin. We conclude that MbfA and Bfr work in concert to manage iron and oxidative stresses. In addition, the need for iron detoxification is not limited to extreme environments, but is also required for normal cellular function.

## Introduction

The ability of bacteria to sense nutrient availability and adapt accordingly contribute to their success in diverse environments. Iron is an essential nutrient required for many cellular processes. Bioavailability of iron is low in aerobic environment because it is oxidized, and therefore insoluble. High affinity iron acquisition systems are expressed under iron limitation to scavenge the metal. Iron can also be toxic, as it catalyzes the generation of reactive oxygen species (ROS). Thus, metal homeostasis must be maintained.

Because of low bioavailability of iron, studies on the maintenance of iron homeostasis have mostly focused on acquisition of the metal. However, recent work has demonstrated bacterial iron export, and has shown it to be important in homeostatic control. MbfA from *Bradyrhizobium japonicum* is an inner membrane CCC1 family protein that is expressed under high iron conditions to export the metal from cytoplasm [1]. An *mbfA* mutant of *B*. *japonicum* or *Agrobacterium tumefaciens* [2] displays elevated iron levels. Derepression of PfeT, a P type ATPase in *Bacillus subtilis*, results in enhanced growth under high iron condition. PfeT catalyses iron export and the ATPase activity is Fe^2+^ dependent [3]. Ectopic expression of MdtD of *Salmonella typhimurium* lowers intracellular iron levels and enhances survival, in cells with elevated iron import [4]. Overproduction of *E*. *coli* FetA and FetB decreases intracellular iron content but no transport studies have been shown in this system [5].

*Bradyrhizobium japonicum* lives as a free-living soil organism or as the endosymbiont of soybean and some other legumes, where it fixes atmospheric nitrogen to ammonia to fulfill the nitrogen requirements of the host. Soils are highly variable ecosystems, and symbiosis represents a niche with specific nutritional requirements. Thus, *B*. *japonicum* and other rhizobia must be able to accommodate changes in metal availability. *B*. *japonicum* belongs to the α-proteobacteria, a large taxonomic group that occupies diverse niches, including within eukaryotic cells in a symbiotic or pathogenic context. *B*. *japonicum* serves as a model system to understand metal metabolism and homeostasis in many α-proteobacterial species [6].

Iron and oxidative stress are intertwined because iron is involved in oxygen chemistry resulting in formation of reactive oxygen species (ROS). In *B*. *japonicum*, an *mbfA* mutant is sensitive to both high iron and peroxide exposure, presumably due to increased available iron content in cells. Similarly, overexpression of MbfA in *Agrobacterium* protects the cell against peroxide mediated killing [7]. FetA and FetB of *E*.*coli* were identified in a screen for cells exhibiting enhanced H_2_O_2_ resistance [5]. In *Salmonella*, expression of MdtABCD operon is induced by nitric oxide stress, and provides resistance to antibiotics that generate ROS [4].

*mbfA* is negatively regulated by Irr, the global iron responsive transcriptional regulator in *B*. *japonicum*. Irr is stable in cells under iron-limited conditions, but degrades in a heme-dependent manner under iron replete conditions [8]. Irr is also degraded upon exposure to H_2_O_2_ [9]. Thus oxidative stress is coordinated with the cellular iron status.

In the present study, we found that an *mbfA* mutant can be resistant to iron and oxidative stresses under certain conditions, and identified the iron storage protein bacterioferritin as the cellular factor responsible for this protection.

## Results

### An *mbfA* mutant grown in high iron medium acquires resistance to iron and hydrogen peroxide stress

**Fig 1:**
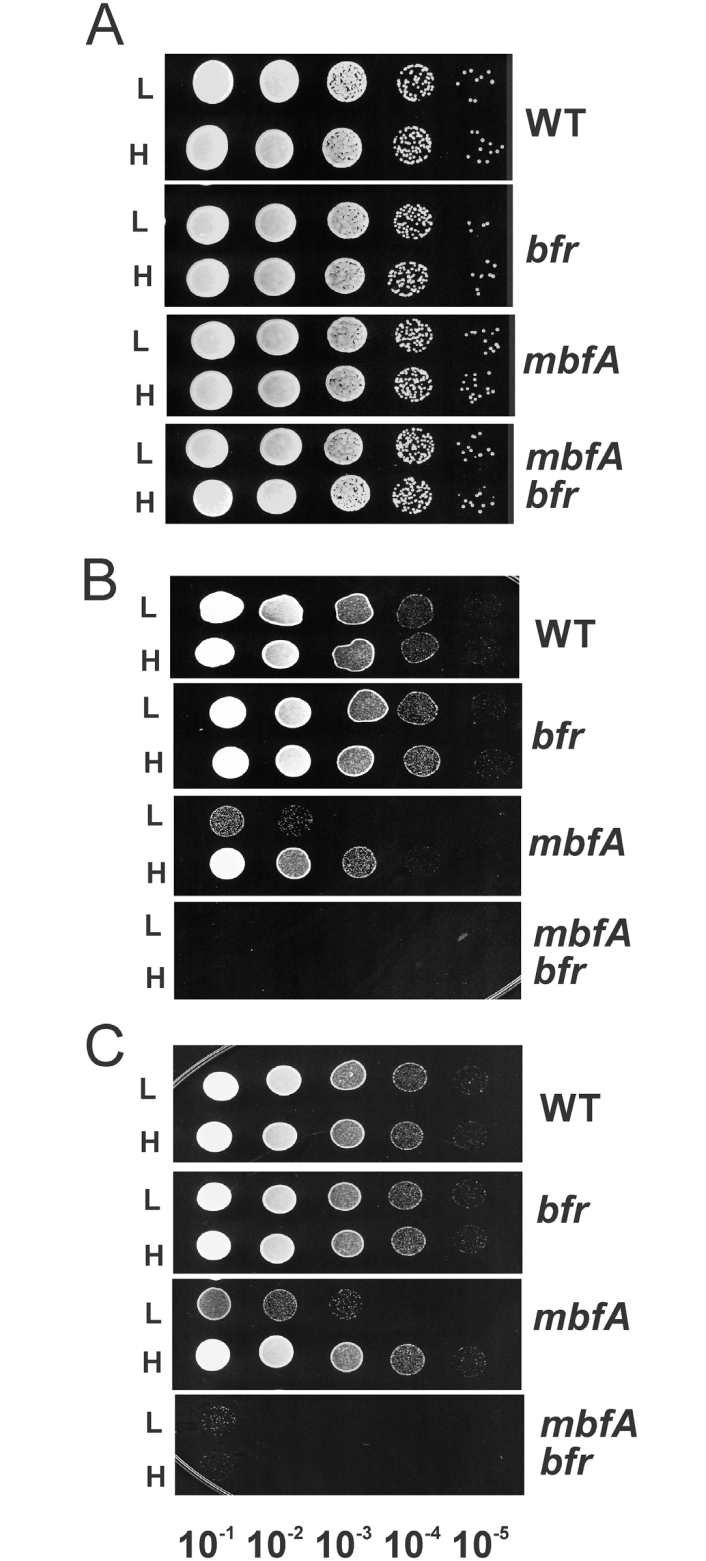
Adaptation to high iron and H_2_O_2_ stress in an *mbfA* strain is lost in a *mbfA bfr* double mutant. Cells of the wildtype, *bfr*, *mbfA*, and *mbfA bfr* strains were grown in medium supplemented with either 3.7 M μM (L) or 100 μM (H) FeCl_3_. Cells grown to mid-log phase were either untreated (A), treated with 5 mM FeSO_4_ (B), or 5 mM H_2_O_2_ (C) for 2 hours. Cells were then serial diluted 10^−1^- to 10^−6^-fold and spotted onto non-selective plates and grown at 29 C. Each panel represents a single plate, and the image was separated for each strain for clarity of presentation.

MbfA is the major iron exporter in *B*. *japonicum*. In a previous study, we demonstrated that an *mbfA* mutant is sensitive to short term (2 hours) exposure to very high iron (5 mM FeSO_4_) when they were first grown in standard growth medium which contains 3.7 μM FeCl_3_ [1]. In the current study, we examined the sensitivity of the *mbfA* mutant to this iron stress after growth in medium containing 100 μM FeCl_3_. Cells of the wildtype and the *mbfA* mutant were grown in medium with 3.7 μM FeCl_3_ or 100 μM FeCl_3_ to mid log phase and then treated with 5 mM FeSO_4_ for 2 hrs or no added iron as a control. Cells were then serial diluted and spotted on non-selective plates to assess cell viability (Fig 1). In contrast to what was observed with cells grown with 3.7 μM FeCl_3_, the *mbfA* mutant was resistant to high iron stress and similar to the wildtype when grown in 100 μM FeCl_3_ (Fig 1B).

We showed previously that the *mbfA* mutant is also sensitive to short term H_2_O_2_ exposure [1]. Here, we compared H_2_O_2_ sensitivity of the wildtype and the *mbfA* mutant strain grown in medium supplemented with 3.7 μM FeCl_3_ or 100 μM FeCl_3_. Similar to the observations with iron stress, the *mbfA* mutant was resistant to a 2 hour exposure to 5 mM H_2_O_2_ treatment, when first grown in medium supplemented with 100 μM FeCl_3_ (Fig 1C). The findings indicate that chronic exposure to an elevated iron level during growth results in adaptation by the *mbfA* mutant to resist iron and H_2_O_2_ toxicity.

### The *bfr* gene encoding bacterioferritin is synthetic lethal with the *mbfA* gene

We wanted to determine what cellular changes occur in response to growth in 100 μM iron that render the *mbfA* strain resistant to iron and oxidative stresses. The *mbfA* gene is co-regulated with the *bfr* gene encoding bacterioferritin, and both genes are derepressed under high (100 μM) iron conditions [10]. Both genes are repressed by the transcriptional regulator Irr, which binds their promoters with over 200-fold greater affinity than other Irr regulon gene promoters. As a result, full derepression of *mbfA* and *bfr* occurs at higher iron concentrations than for other Irr-repressed genes [10]. In addition, the *bfr* gene is expressed more highly in an *mbfA* strain than the wild type when grown in media supplemented with 100 μM iron [1]. Thus, it is plausible that bacterioferritin has an iron storage capacity that can compensate for the iron export defect when cells are grown in high iron media.

To test this, we constructed an in-frame deletion of the *bfr* gene in both wild type and *mbfA* mutant backgrounds, and tested the sensitivity of the mutants to exposure to high iron and H_2_O_2_ exposure. The *bfr* mutant had a similar phenotype as the wild type with respect to viability in response to a 2 hour exposure to 5 mM FeSO_4_ or 5 mM H_2_O_2_ when first grown under 3.7 μM or 100 μM iron conditions (Fig 1B and 1C). However, the *mbfA bfr* double mutant was extremely sensitive to exposure to high iron or H_2_O_2_ (Fig 1A and 1B). The synthetic lethality of the *bfr* gene with *mbfA* suggests that elevated *bfr* expression caused by growth in 100 μM iron confers resistance to iron and H_2_O_2_ in the *mbfA* mutant strain. Interestingly, the double mutant was also sensitive to these stresses when grown in normal iron media, suggesting that bacterioferritin mitigates the effects of the MbfA defect under that condition even though *bfr* is expressed at a lower level.

### Overexpression of the *bfr* gene rescues *mbfA* mutant phenotypes without elevating iron in the growth medium

**Fig 2:**
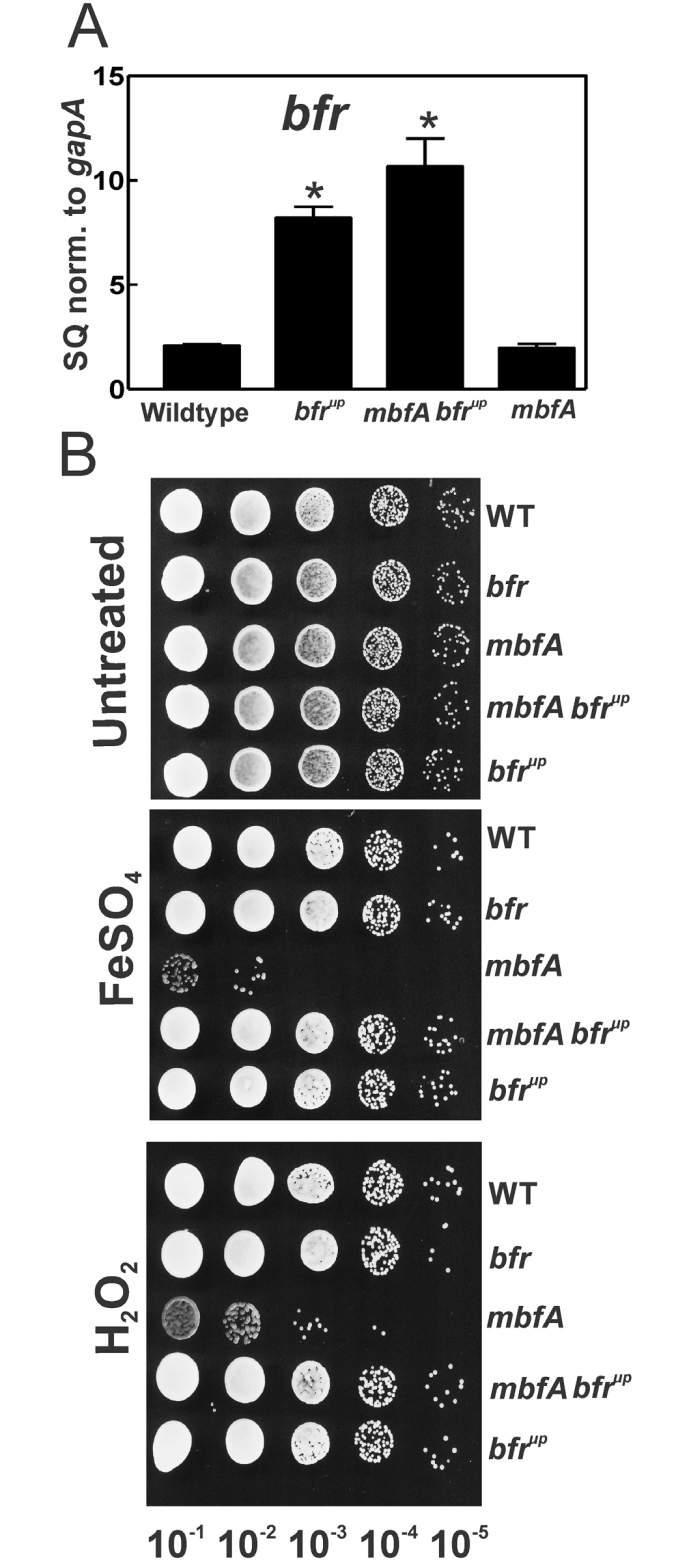
Rescue of *mbfA* mutant phenotypes by *bfr* overexpression. A) Steady state transcript levels of the *bfr* gene in the wildtype, *bfr*^*up*^, *mbfA bfr*^*up*^ and *mbfA* grown in medium with 3.7 M μM FeCl_3_ were analyzed by quantitative real-time PCR. The data are expressed as relative starting quantities (SQ) of respective mRNAs normalized to the house keeping gene *gapA*, and presented as average of three replicates ±SD. An asterisk denotes a significant difference compared to the wild type based on Student’s *t* test using a confidence level of *p<*0.01. B) Cells of the *bfr*^*up*^, *mbfA bfr*^*up*^ and *mbfA* were grown in medium supplemented with 3.7 μM FeCl_3_. Cells grown to mid log phase were either untreated, treated with 5 mM FeSO_4_ or 5 mM H_2_O_2_ for 2 hours. Cells were then serial diluted 10^−1^ to 10^−6^ fold and spotted onto non selective plates.

If up regulation of the *bfr* gene in response to growth in 100 μM Fe is responsible for rescuing the *mbfA* mutant phenotypes, then elevating *bfr* expression by some other method should yield similar results even at lower iron concentrations. The *bfr*^*up*^ gene contains a modified promoter that binds Irr with lower affinity [10]. As a result, the *bfr* gene is more derepressed at lower iron concentrations, which was confirmed here by measuring *bfr* mRNA by qPCR in the wild type and *bfr*^*up*^ strains. (Fig 2A). An *mbfA bfr*^*up*^ mutant was constructed, and expression of the *bfr* gene was elevated relative to the wild type (Fig 2A). Cells grown in GSY media (3.7 μM Fe) were analyzed for sensitivity to high iron or H_2_O_2_ exposure. The *mbfA bfr*^*up*^ mutant was much more resistant to H_2_O_2_ or high iron exposure than the *mbfA* mutant, and was similar to the wild type (Fig 2B). These findings show that elevated *bfr* levels are sufficient to rescue *mbfA* mutant phenotypes, and further show a functional relationship between the two gene products.

### The *mbfA bfr* double mutant has a severe growth phenotype under high iron conditions

**Fig 3:**
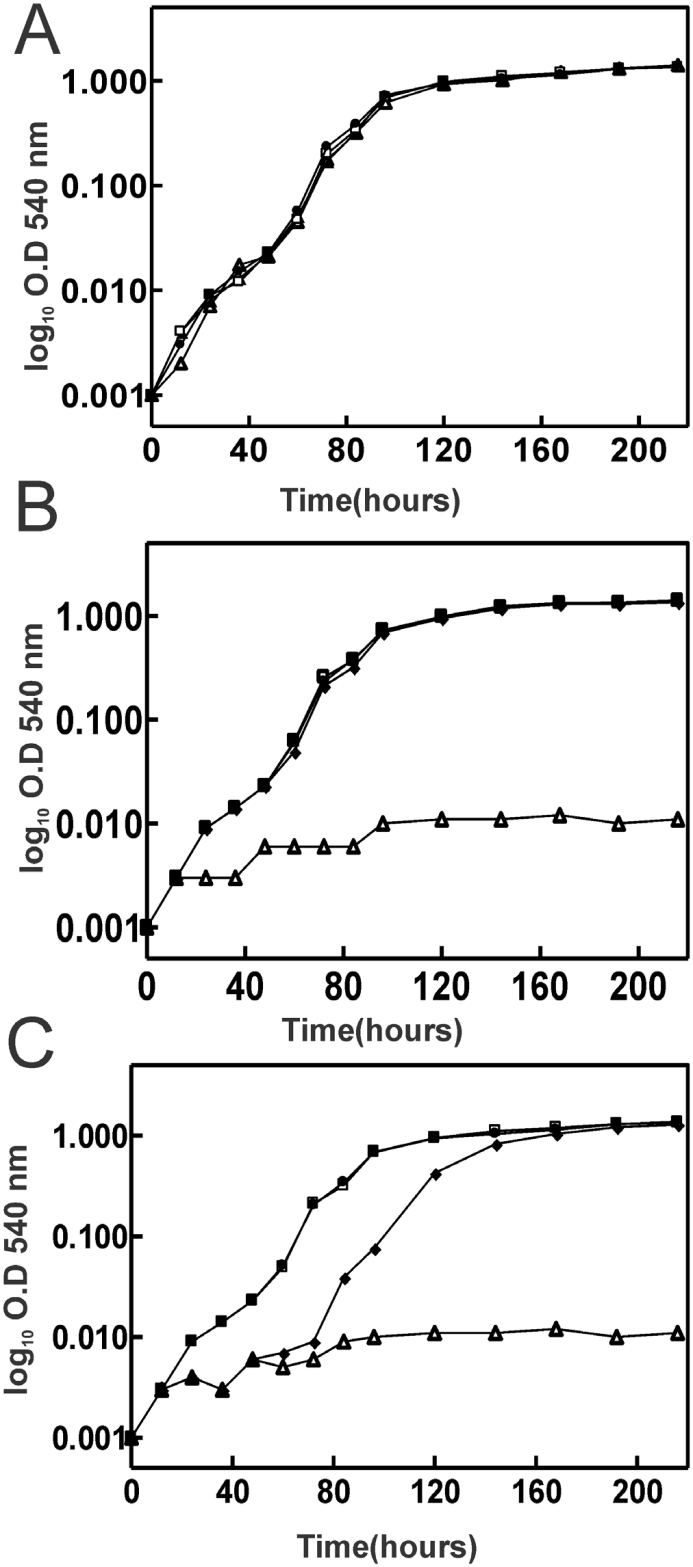
Growth curves of wild type and mutant strains under various iron conditions. Growth media were inoculated with 1 × 10^6^ cells/ml of the wildtype (closed circles), *bfr* (open squares) *mbfA* (closed diamonds) and *mbfA bfr* (open triangles) strains. Strains were grown in GSY medium supplemented with either 5 μM FeSO_4_ (A), 0.75 mM FeSO_4_ (B), or 1.2 mM FeSO_4_ (C). Aliquots were taken at the indicated time points and the optical density was measured at 540 nm (OD_540_). The time points are the average of three biological replicates ± the standard deviation.

We established that MbfA and Bfr are involved in managing acute exposure to iron and H_2_O_2_. We wanted to establish their role in chronic iron stress during growth in liquid medium. We carried out the growth experiments with FeSO_4_ and sodium citrate rather than FeCl_3_ to maintain solubility of iron at high concentrations. When grown in media supplemented with 5 μM FeSO_4_, the *mbfA* and *bfr* single mutants and the *mbfA bfr* double mutant grew similarly to the wild type (Fig 3A). The wild type also grew well in media containing 750 μM FeSO_4_ (Fig 3B), as did the *mbfA* and *bfr* single mutants. However, the *mbfA bfr* double mutant had a severe growth phenotype under high iron conditions. Thus, MbfA and Bfr are involved in coping with growth under high iron conditions, and each protein can compensate for a defect in the other to maintain normal growth. When grown in medium supplemented with 1.2 mM FeSO_4_, the *bfr* mutant grew well, but the *mbfA* mutant displayed a growth defect. Thus, at this iron concentration, MbfA is required for normal growth, and Bfr activity is not sufficient to detoxify iron.

### MbfA and Bfr are required to maintain iron homeostasis under moderate iron conditions

Thus far, we have examined extreme environmental conditions to assess an equally extreme phenotype, namely cell viability. We wanted to address the roles of MbfA and Bfr under moderate iron conditions, where the mutants remain viable. We examined intracellular iron content in the wild type and mutant cells by ICP-MS grown in media supplemented with no or 5 μM iron. We used FeSO_4_ rather than FeCl_3_ for these experiments because we found that the ICP-MS data were more reproducible with FeSO_4_, possibly due to better solubility.

**Fig 4:**
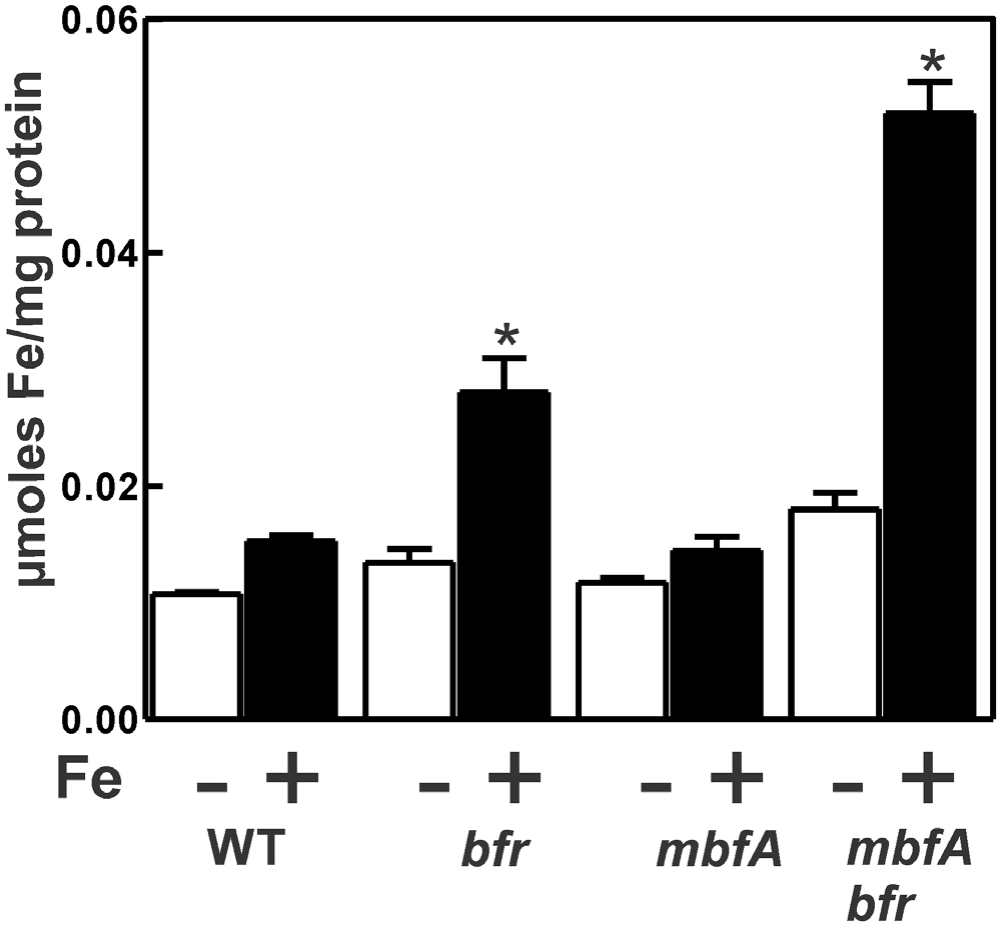
Total intracellular iron content wild type and mutant strains. Cells of the wildtype, *bfr*, *mbfA* and *mbfA bfr* strains were grown in GSY medium supplemented with either no added iron (o*pen bars*) or 5 μM FeSO_4_ (c*losed bars*). Total amounts of Fe were determined by Inductively Coupled Plasma-Mass Spectrometry analysis. The values represented are an average of three runs ± the S.D. An asterisk denotes a significant difference compared to the wild type based on Student’s *t* test using a confidence level of *p<*0.01.

Under low iron conditions, the iron content of the *mbfA* and *bfr* mutants was similar to the wild type, and that of the double mutant was slightly higher (Fig 4). When grown with 5 μM iron, the intracellular iron level in the *mbfA* strain remained low, similar to the wild type. This observation is consistent with the fact that the *mbfA* gene is not derepressed at this iron range [10], which was confirmed here (S1 Fig). The *mbfA bfr* double mutant contained over 3-fold more iron than the wild type or *mbfA* strain, showing that both gene products are involved in maintaining homeostasis, and that they can compensate for each other. Although the *bfr* strain contained less iron than the double mutant, it was greater than was found in the wild type. We cannot yet offer an explanation for this.

### MbfA and Bfr are necessary for normal iron-responsive gene expression

**Fig 5:**
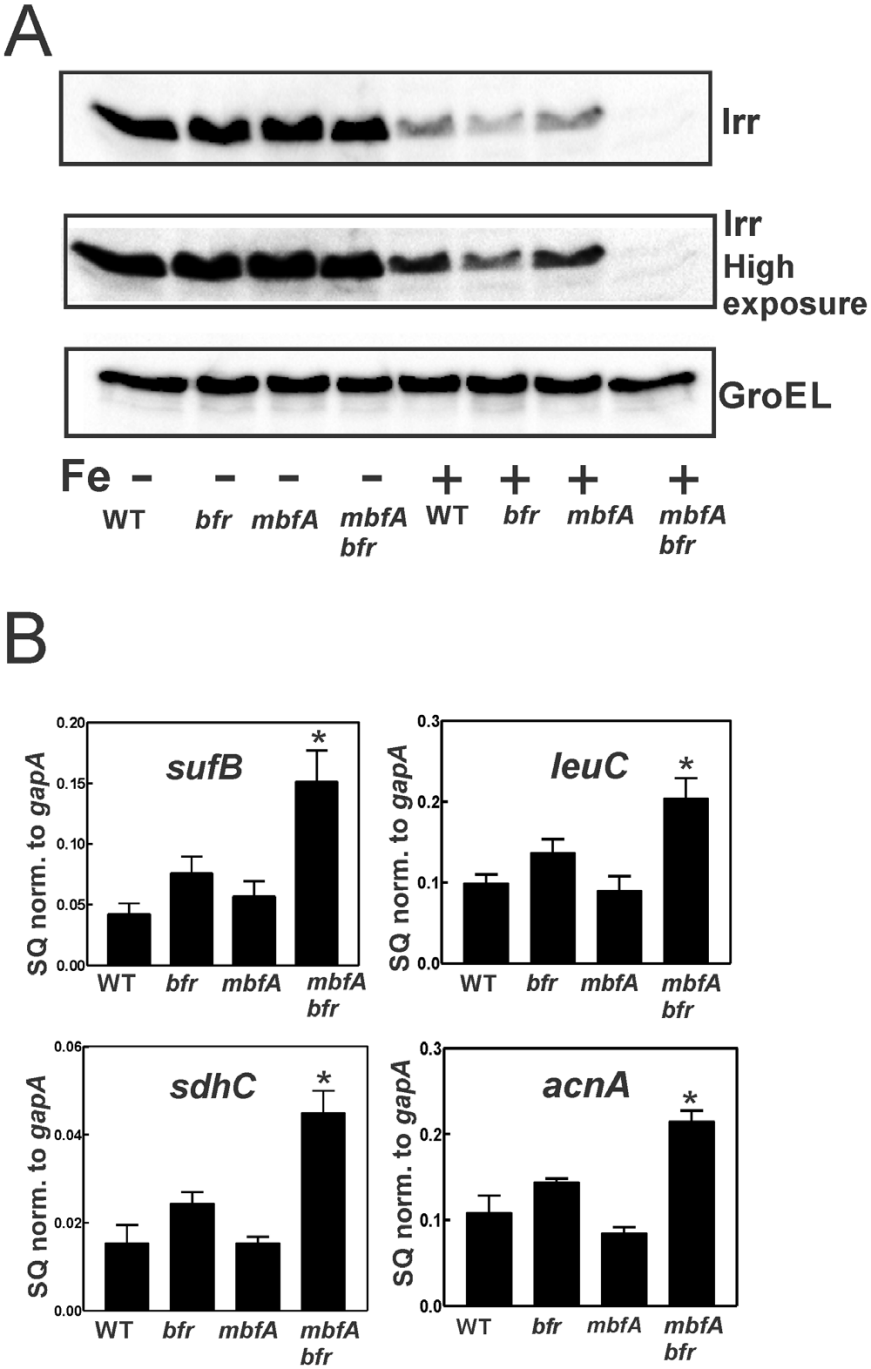
Aberrant regulation of iron-dependent gene expression. A)Western blot analysis of Irr was performed on wildtype, *bfr*, *mbfA* and *mbfA bfr* strains grown in GSY medium supplemented either with no added iron (-) or 5 μM FeSO_4_ (+). The protein was detected using anti-Irr antibodies. The same blot was exposed for either 20 secs (upper panel) or 100 secs (Irr long), lower panel). GroEL was used as a control for a protein not regulated by iron, and it was detected using anti-GroEL antibodies. 15 μg of protein were loaded per lane. B) Cells of the wildtype, *bfr*, *mbfA* and *mbfA bfr* strains were grown in GSY medium supplemented with 5 μM FeSO_4_. The steady-state transcript levels of *leuC*, *sdhC*, *acnA* and *sufC* were analyzed by qualitative real-time PCR. The data are expressed as relative starting quantities (*SQ*) of respective mRNAs normalized to the housekeeping gene *gapA* and are presented as average of three replicates ± S.D (*error bars*). An asterisk denotes a significant difference compared to the wild type based on Student’s *t* test using a confidence level of *p<*0.01.

Because the *mbfA bfr* strain had higher iron content when grown in 5 μM iron, we wanted to determine whether iron-responsive gene expression was affected in the double mutant. Irr is the global iron-responsive transcriptional regulator in *B*. *japonicum* and related bacteria [11]. Irr accumulates under iron limitation, but degrades in response to the metal ([12] and Fig 5A). We monitored Irr levels by Western blot analysis in cells of the wild type, *mbfA*, *bfr* and *mbfA bfr* strains grown in media supplemented with 0 or 5 μM FeSO_4_ (Fig 5A). Irr levels were high in all strains grown under low iron conditions. Wild type cells grown with 5 μM FeSO_4_ contained low but detectable levels of Irr, which was also observed for the *bfr* and *mbfA* single mutants. However, Irr was nearly undetectable in the *mbfA bfr* double mutant, even upon long exposure of the blot. This suggests that the total iron content correlates with the regulatory pool that controls Irr content.

The consequences of very low Irr levels in the *mbfA bfr* double mutant strain were readily observable in expression of Irr-regulated genes. *sufA*, *sdhC*, *leuC* and *acnA* are genes repressed by Irr [1]. mRNA levels of these genes were 2- to 3-fold higher in the *mbfA bfr* double mutant than was observed in the wild type, and also higher than was found in either of the single mutants (Fig 5B). We note that in a previous study [1], the *mbfA* mutant displayed both high iron content and aberrant gene expression. However, in that study, cells were grown in 20 μM or 100 μM iron rather than the 5 μM used here. The observations reinforce the conclusion that iron export and storage work in concert for normal iron-responsive gene expression.

### The *mbfA bfr* double mutant is sensitive to the iron activated antibiotic streptonigrin

**Fig 6:**
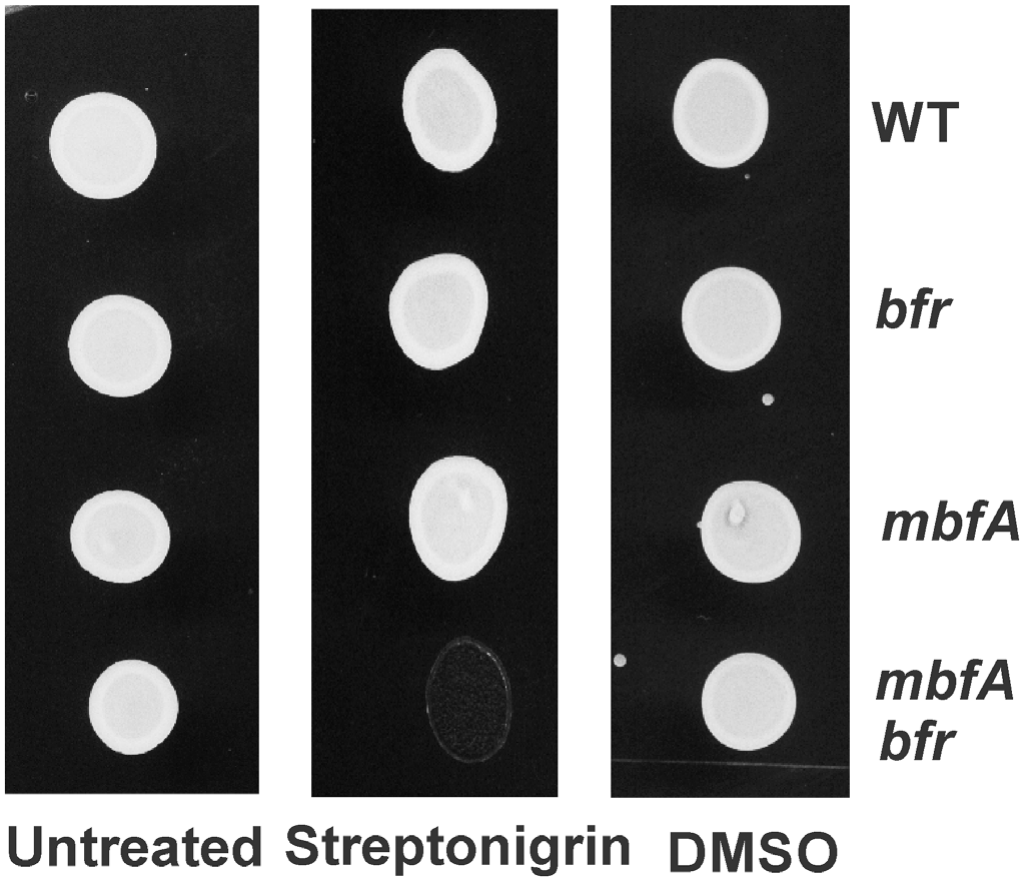
Sensitivity of the double mutant strain to streptonigrin: Cells of the wildtype, *bfr*, *mbfA* and the *mbfA bfr* strains were grown in GSY medium supplemented with 5 μM FeSO_4_ and treated with either 200 μg/ml of streptonigrin, or equal volume of DMSO for 26 hours. Cells were then spotted on non-selective plates. Untreated cells are spotted as an additional control.

Streptonigrin is a quinone containing antibiotic, whose antimicrobial activity is affected by intracellular iron availability [13]. When grown in medium with 5 μM FeSO_4_ and then treated with streptonigrin (dissolved in DMSO), the *mbfA bfr* double mutant was highly susceptible to killing compared to the wildtype or the single mutants (Fig 6). Cells treated with equal volume of DMSO did not show any difference in sensitivity. This shows the compensatory role of Bfr and MbfA in maintaining the iron content and in turn protection against antimicrobial activity of streptonigrin.

### Evidence for interaction between Bfr and the N-terminal domain of MbfA

**Fig 7:**
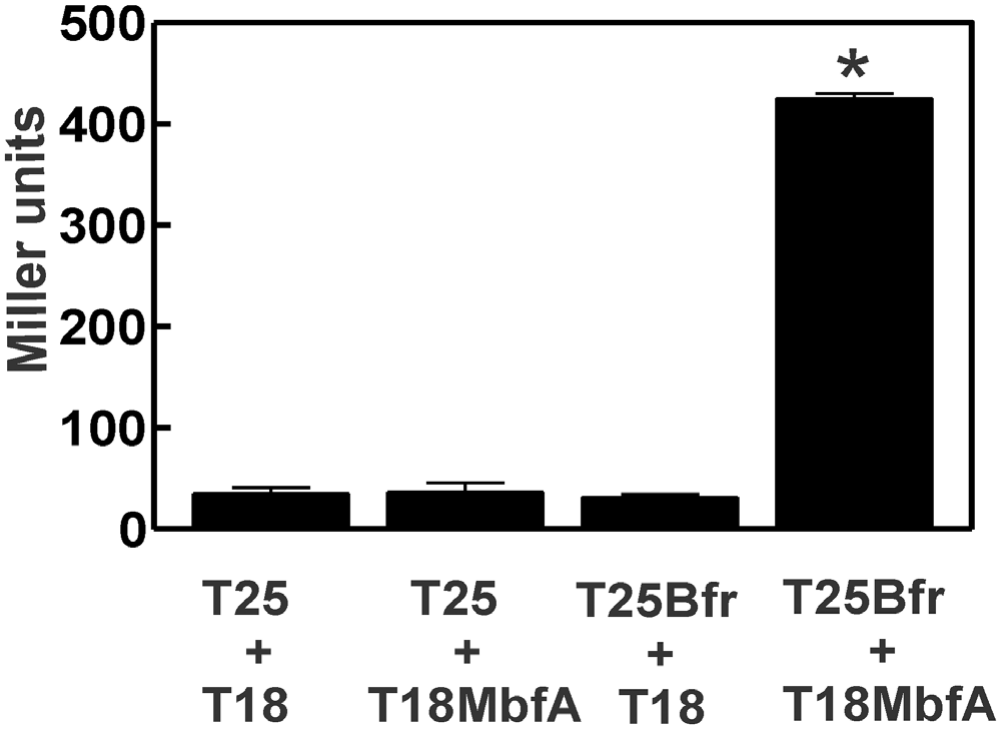
Interactions between FLD of MbfA and Bfr in a bacterial two hybrid assay. β-galactosidase assay was performed on midlog phase grown cells of BTH101 cells harboring pKT25 and pUT18C (T25 and T18), pKT25 and pUT18C-MbfA (T25 and T18MbfA), pUT18C and pKT25-Bfr (T25Bfr and T18) and pKT25-Bfr and pUT18C-MbfA (T25Bfr and 18MbfA). The values represented are an average of three samples± the S.D. (*error bars*). An asterisk denotes a significant difference compared to the control containing the empty vectors pKT25 and pUT18C based on Student’s *t* test using a confidence level of *p<*0.01.

MbfA has an N-terminal ferritin-like domain (FLD) that dimerizes in solution [1]. Similarly, bacterioferritin functions as an oligomer. This raises the possibility that MbfA and Bfr physically interact with each other at their respective ferritin-like domains. To address this, a bacterial two hybrid assay was carried out [14] in which Bfr and the MbfA FLD were expressed as fusion proteins with domains of adenylate cyclase. Interactions restore adenylate cyclase activity, which was measured as β-galactosidase activity (Fig 7). Expression of one fusion protein alone in *E*. *coli* strain BTH101 yielded very low activity, similar to the strain harboring vectors without inserts. However, expression of T25-Bfr and T18-MbfA together resulted in high β-galactosidase (Fig 7). These finding suggest that Bfr and MbfA interact with each other.

## Discussion

In the present study, we found that an iron export mutant of *B*. *japonicum* can adapt to brief exposure to very high levels of iron or H_2_O_2_ by elevated expression of the *bfr* gene encoding bacterioferritin. The synthetic lethality of *mbfA* with *bfr* argues in favor of a functional relationship between their gene products. It is also consistent with the coordinate control of the two genes by the high affinity of Irr for each promoter, leading to maximal derepression when iron or H_2_O_2_ levels are elevated.

A functional link between the export and storage of iron makes sense because both activities presumably reduce active iron in cells. The physical properties of bacterioferritin are well-described [15], but less is known about its role in cells. Bacterioferritin is part of the ferritin family of proteins that also includes Ferritin and Dps. These proteins are dissimilar at the amino acid sequence levels, but share similar architecture, including the ability to oligomerize. Many bacteria contain more than one ferritin family protein, which may hamper the elucidation cellular roles due to functional redundancy. *Neisseria gonorrhea* appears to have only bacterioferritin, and a *bfr* mutant is sensitive to oxidative stress [16], whereas *E*. *coli* also has ferritin, and a *bfr* mutant does not have discernible phenotypes [17]. Iron taken up by *Erwinia chrysanthemi* is found in bacterioferritin, shortly after uptake, but little remains there after 40 minutes [18], suggesting that the protein does not serve as a long term iron reserve. The current work shows clearly that bacterioferritin participates in managing iron and oxidative stresses. However, the weak phenotype of a *bfr* mutant appears not to be due to compensatory activities of the other putative ferritin family protein in the *B*. *japonicum* genome (*bll7374* and *bll0290*), but rather to iron export activity.

A *B*. *subtilis* iron export mutant cannot grow in media containing 4 mM iron, but that phenotype is partially suppressed by a second mutation in in the gene encoding the transcriptional repressor PerR [3]. The Dps protein MrgA is derepressed in the *perR* strain, which rescued the export mutant. Thus, even though *B*. *japonicum* and *B*. *subtilis* have different exporters and iron storage proteins, the activities are functionally linked, suggesting a broadly applicable bacterial theme.

*B*. *japonicum* grows well in liquid media containing 750 μM iron, and our findings show that MbfA or Bfr can manage the high iron level without the other, as observed by the robust growth in a *bfr* or *mbfA* mutant, respectively (Fig 3B). The severe growth phenotype of *mbfA bfr* double mutant under high iron growth indicates that there is not an additional compensatory mechanism. The *B*. *japonicum* genome harbors genes encoding other putative ferritin family proteins, but they appear not to play a role under that condition. When the iron content in the media was elevated to 1.2 mM, the *mbfA* mutant had a growth phenotype, but the *bfr* mutant did not (Fig 3C). It is likely that the storage protein saturates with iron and reaches a threshold where it can no longer protect cells from iron stress. The effectiveness of MbfA, however, should be limited by the rate at which it can export, and the good growth of the *bfr* strain even in 1.2 mM iron indicates that MbfA activity is sufficient to maintain viability. Within symbiotic soybean root nodules, it is estimated that the medium surrounding *B*. *japonicum* cells ranges from 0.5 mM to 2.5 mM [19]

Although the *mbfA bfr* mutant had a growth phenotype under high iron or H_2_O_2_ exposure, it also displayed phenotypes in a modest iron environment. In particular, the double mutant had elevated iron levels, increased sensitivity to the antibiotic streptonigrin and aberrant iron-responsive gene expression (Figs 4, 5 and 6). These observations show that the need for iron detoxification is not limited to extreme environments, but instead, it is likely to be integral to maintaining iron homeostasis more broadly.

## Materials and Methods

### Strains and Media

*B*. *japonicum* USDA110 is the parent strain used in this study. *B*. *japonicum* strains were routinely grown at 29°C in GSY medium as described previously [20]. The actual iron concentration of the unsupplemented medium was 0.3 μM, as determined with a PerkinElmer Life Sciences model 1100B atomic absorption spectrometer.

### Construction of strains and plasmids

For creating the *bfr* mutant, the open reading frame plus 600 bp of flanking DNA was isolated by PCR using genomic DNA as a template and ligated into pBluescript SK2. A deletion removing only the open reading frame was constructed by inverse PCR as described previously [21], and the product was blunt ligated. The resulting flanks were then restriction digested from pBluescript SK2, ligated into the vector pLO1 and introduced into *B*. *japonicum* USDA110 by conjugation. Single recombinants were selected based on kanamycin resistance and then screened for sensitivity on growth with 5% (wt/vol) sucrose due to the *sacB* gene harbored on the plasmid. Double recombinants arising from a selected single recombinant were selected based on sucrose resistance and then screened for kanamycin sensitivity. The *bfr* deletion in the wildtype was confirmed by the size and sequence of the PCR products using primers in the flanks. The same pLO1 vector was introduced to *mbfA* mutant background and the same selection criteria was employed to create *mbfA bfr* double mutant. A pLO1 strain with ICE motif in promoter of *bfr* substituted by ICE motif of *fhuE* was made in a previous study [10]. This construct was moved into the *mbfA* mutant background, and the same selection criteria of single and double recombinants were made to make *mbfA bfr*^*up*^.

### Iron and H_2_O_2_ Sensitivity Assays

Cells were grown in either 3.7 μM FeSO_4_ or 100 μM FeSO_4_ up to mid log phase and treated with either 5 mM FeSO_4_ or 5 mM H_2_O_2_ for 2 hours. Cells were then washed and serially diluted in PBS. The serial dilutions were then spotted on regular GSY plates.

### Growth curve analysis

Cells were grown in GSY medium with either 3.7 μM FeSO_4_, 0.75 mM FeSO_4_ or 1.2 mM FeSO_4_ as described in the text. In order to avoid formation of insoluble ferric hydroxides 1 g/l of citrate trisodium dihydrate was added to the GSY medium. Growth rates were analyzed by measuring the optical density of cells at 540 nm at given time intervals until they reached stationary phase.

### Western Blot Analysis

Cells were harvested by centrifugation at 13,000×g for 7 min, washed twice in phosphate-buffered saline (10 mM Na_2_HPO_4_, 2 mM KH_2_PO_4_, 137 mM NaCl, and 2.7 mM KCl, pH 7.4), and resuspended in the same buffer. The protein concentrations were measured by the BCA protein assay (Pierce). 15 μg of protein from each sample were boiled in SDS loading buffer and loaded on 15% polyacrylamide gel, and immuno blotting was carried out. Anti-Irr antibody was used at a dilution of 1:2500. Anti-GroEL (Enzo Life Sciences) was used at a dilution of 1:8000. HRP-conjugated goat anti-rabbit IgG (SouthernBiotech, Birmingham, AL) was used as secondary antibody, and the blot was detected using the Immobion chemiluminescence system (Millipore).

### Quantitative real time PCR

Cells were grown to mid log phase, and RNA was isolated by the hot phenol method as described previously [11]. 1μg of RNA from each strain were used to make cDNA using Bio-Rad cDNA synthesis kit. Quantitative PCRs were performed as described previously [11]. Data were normalized to *gapA* and are expressed as average of triplicates, with S.D. represented by the *error bars*.

### Metal Content Determination

40 ml of cells were grown in GSY with either no added iron or 5 μM FeSO_4_. The cells were harvested at mid log phase by centrifugation at 13,000×g for 5 min. The pellets were washed twice with ice-cold phosphate buffered saline (PBS) buffer containing 0.1 M EDTA. To remove excess salt, the pellets were washed twice with metal free PBS. Samples were centrifuged and 10 μl were removed for protein estimation by Bradford assay. To lyse the cells completely, the pellets were treated with 100 μl of 70% nitric acid and incubated at 98°C for 3 h. 1 ml of double-distilled metal free water were added to this preparation and centrifuged at 13,000×g for 5 min to remove cell debris. The supernatant was sent for inductively coupled plasma-Mass Spectrometry analysis (Penn State Institutes of Energy and the Environment, State college, Pennsylvania).

### Streptonigrin sensitivity assay

Streptonigrin was dissolved in DMSO to make a stock of 10 mg/ml. Cells were grown to mid log phase and Streptonigrin, at a final concentration of 200 μg/mL was added to the medium. Cells were treated with equal volume of DMSO as a control. After 26 hours, cells were collected and spotted on regular GSY plates.

### Bacterial two hybrid assay

A bacterial two hybrid system was used as described previously [14] to monitor interactions between Bfr and the N-terminal ferritin-like domain (FLD) of MbfA.

The open reading frame of the *bfr* gene was ligated into pKT25 to construct a gene encoding a fusion protein of the N-terminal portion adenylate cyclase with Bfr. The FLD ORF was ligated into pUT18C to construct a gene encoding a fusion protein of FLD with the C-terminus of adenylate cyclase. Interaction between the proteins restores adenylate cyclase activity in cells, resulting in cyclic AMP synthesis and activation of the transcriptional regulator CAP. This, in turn, activates the β-galactosidase gene. β-galactosidase activity was measured in *E*. *coli* BTH101 cells harboring the plasmids as described [22]. Cells were grown aerobically in LB medium at 30°C until they reached mid log phase. Cells were spun down and resuspended in 800 μl Z buffer (60 mM Na_2_HPO_4_ 7H_2_O, 40 mM NaH_2_PO_4_.H_2_O, 10 mM KCl, 1 mM MgSO_4_ 7H_2_O and 50 mM β-mercaptoethanol, pH 7·0). One hundred microlitres of suspension, corresponding to 1×10^8^ cells, was used per reaction. The data are represented in Miller units and each value is a mean of triplicate samples corrected for background. Absorbance of *o*-nitrophenol formed from ONPG by β-galactosidase was recorded at 420 nm and normalized for cell density at OD_550_.

## Supporting Information

S1 Fig Steady state transcript levels of *mbfA* or *bfr* gene mRNA in wild type cells. (PDF)
