# Supplementary material for: Synthetic Lethality of the bfr and mbfA Genes Reveals a Functional Relationship between Iron Storage and Iron Export in Managing Stress Responses in Bradyrhizobium japonicum
Source: PLoS One. 2016 Jun 10;11(6):e0157250. doi: 10.1371/journal.pone.0157250 (PMC4902296; doi:10.1371/journal.pone.0157250)
Supplement: S1 Fig — (PDF) [file pone.0157250.s001.pdf]

Supporting Information

Sankari, S and O'Brian, M.R.

Synthetic lethality of the *bfr* and *mbfA* genes reveals a functional relationship between iron storage and iron export in managing stress responses in *Bradyrhizobium japonicum*

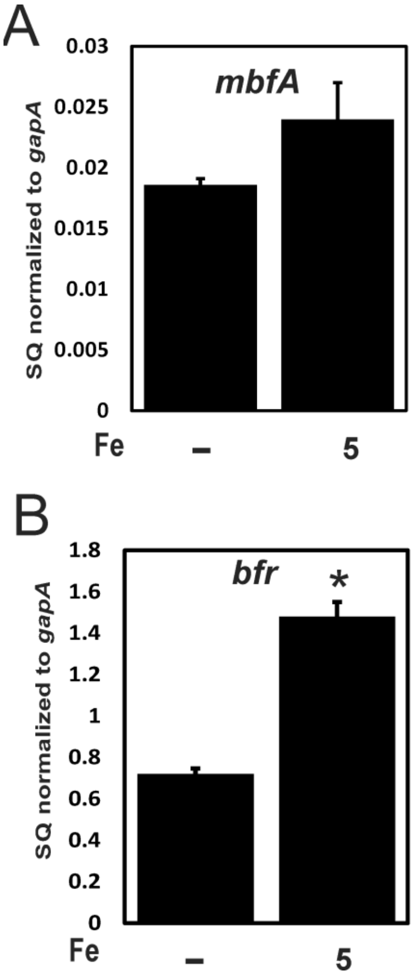

Figure S1. Steady state transcript levels of *mbfA* (A) or *bfr* (B) gene mRNA in wild type cells. Cells were grown in media containing no added iron (-) or 5 μM $FeSO_4$. RNA was analyzed by quantitative real-time PCR. The data are expressed as relative starting quantities (SQ) of respective mRNAs normalized to the house keeping gene *gapA*, and presented as average of three replicates ±SD. An asterisk denotes a significant difference in the mRNA level in cells grown in 5 μM iron compared to that grown in media without iron supplementation based on Student's *t* test using a confidence level of $p<0.01$.
